# Study of the Influence of the Orientation of a 50-Hz Magnetic Field on Fetal Exposure Using Polynomial Chaos Decomposition

**DOI:** 10.3390/ijerph120605934

**Published:** 2015-05-27

**Authors:** Ilaria Liorni, Marta Parazzini, Serena Fiocchi, Paolo Ravazzani

**Affiliations:** 1 CNR Consiglio Nazionale delle Ricerche, Istituto di Elettronica e di Ingegneria dell’Informazione e delle Telecomunicazioni IEIIT, Piazza Leonardo da Vinci 32, Milan 20133, Italy; E-Mails: marta.parazzini@ieiit.cnr.it (M.P.); serena.fiocchi@ieiit.cnr.it (S.F.); paolo.ravazzani@ieiit.cnr.it (P.R.); 2 Dipartimento di Elettronica, Informazione e Bioingegneria DEIB, Politecnico di Milano, Piazza Leonardo da Vinci 32, Milan 20133, Italy

**Keywords:** fetus, ELF-MF exposure, stochastic dosimetry, polynomial chaos

## Abstract

Human exposure modelling is a complex topic, because in a realistic exposure scenario, several parameters (e.g., the source, the orientation of incident fields, the morphology of subjects) vary and influence the dose. Deterministic dosimetry, so far used to analyze human exposure to electromagnetic fields (EMF), is highly time consuming if the previously-mentioned variations are considered. Stochastic dosimetry is an alternative method to build analytical approximations of exposure at a lower computational cost. In this study, it was used to assess the influence of magnetic flux density (**B**) orientation on fetal exposure at 50 Hz by polynomial chaos (PC). A PC expansion of induced electric field (**E**) in each fetal tissue at different gestational ages (GA) was built as a function of **B** orientation. Maximum **E** in each fetal tissue and at each GA was estimated for different exposure configurations and compared with the limits of the International Commission of Non-Ionising Radiation Protection (ICNIRP) Guidelines 2010. PC theory resulted in an efficient tool to build accurate approximations of **E** in each fetal tissue. **B** orientation strongly influenced **E**, with a variability across tissues from 10% to 43% with respect to the mean value. However, varying **B** orientation, maximum **E** in each fetal tissue was below the limits of ICNIRP 2010 at all GAs*.*

## 1. Introduction

The exposure to electromagnetic fields (EMF) is day by day of growing interest due to the public concern over its possible health effects. Although this situation has lasted for many decades, the correct and quantitative assessment of the human exposure to EMF still represents an urgent priority [1].

The assessment of human exposure consists of the quantification of the induced electric field or the electric current density in the low-frequency (LF) range and of the quantification of the specific absorption rate (SAR) in the radio frequency (RF) range. These metrics are used on one side by international regulatory bodies, which have established exposure guidelines to prevent all of the known short-term health effects [2,3]. On the other side, they are applied by health risk assessors to estimate the EMF exposure in order to determine the health impact of EMF related to a specific health risk [4]. 

In the evaluation of human exposure to EMF, it is necessary to take into account several parameters, which vary in a real exposure scenario and influence the dose, *i.e*., the induced electric field and electric current density at LF and the SAR at RF [5]. As an example of these parameters, one should consider the location, the frequency band of the source and the incident field orientation, but also parameters directly related to the subject exposed, such as the morphology and the dielectric properties of tissues, which could change with age [6,7,8,9], and also the posture of the subject with respect to the incident EMF field. 

Typically, the estimation of dosimetric quantities is performed through deterministic dosimetry, which solves the electromagnetic problem by means of computational electromagnetics techniques, such as Finite-difference time-domain (FDTD) method and Finite element method (FEM). Even though deterministic dosimetry is a helpful tool to assess the human exposure to EMF, when the variation of the parameters previously introduced is taken into account, it could lead to highly time-consuming simulations, even though acceleration has been realized in recent years [10], one simulation being necessary for each specific exposure condition. However, more realistic exposure assessments, passing from “one-case” estimation to the evaluation of real‑life exposure conditions, are, day by day, considered inalienable, and their introduction cannot be delayed anymore. 

Stochastic methods applied to EMF dosimetry sound like promising techniques, capable of providing the assessment of the exposure in realistic conditions at a lower computational cost than the one necessary with deterministic dosimetry. In general terms, the application of these mathematical methods to assess the variability of EMF exposure is called stochastic dosimetry.

There are many stochastic methods that can be used for that purpose. Their common aim is to build a model of the system under investigation on the basis of a few observations of the system output Y obtained by computational methods or based on experimental measurements. In the specific case of the EMF analysis, the system output Y is the exposure quantity under evaluation. The inputs of the model are the parameters x_i_ collected in the input random vector **X**, whose changes and consequent effect on the system output Y are the goal of the investigation. 

As examples of these stochastic methods, the Monte Carlo approach presents a slow convergence rate and requires a high number of simulations to reach a satisfactory accuracy. Therefore, this approach is intractable in the case of computationally demanding models [5,11], such as the ones used in EMF dosimetry. 

On the other side, spectral methods represent an approximation of the system output Y on a suitable functional basis [12,13]. One of them, called polynomial chaos (PC) [13,14,15], consists of using orthogonal polynomials as the functional basis. 

In the literature, there exist few studies in which PC theory is applied to analyze human exposure to EMF [5,16,17,18]. In [5], PC theory is applied to the analysis of the influence of the direction of arrival of a plane wave on the fetal exposure to RF. Voyer and colleagues [16] discussed different methods of using the PC approach for 2D computational electromagnetics, by which the computational cost can be reduced. In [17], exposure to mobile phones is analyzed, building a PC expansion of the SAR in the head of the exposed subject, in order to estimate the influence of the position of the mobile phone relative to the head on the SAR. In [18], PC theory is used to quantify the variability of eddy currents in the adult brain at LF, changing the dielectric properties of brain tissues. Furthermore, in [19], polynomial chaos has been used in a biomedical application to assess the influence of the uncertainty of the material properties of brain tissue at a frequency of 2 kHz on the probabilistic voltage response and the probabilistic volume of tissue activated (VTA) in a FEM volume conductor model of deep brain stimulation. 

In this study, an approach based on PC theory has been applied to assess the variability of human exposure at LF. In detail, the influence of the incident **B**-field orientation at 50 Hz has been estimated on the fetal exposure at different stages of pregnancy. So far, several studies in the literature have analyzed the fetal exposure at 50 Hz by means of deterministic dosimetry with appropriate anatomical modelling [20,21,22,23,24]. In [20], models of the embryo and fetus at 8, 13, 26 and 38 weeks of pregnancy were combined with an anatomically realistic adult female model [25]. The whole-body fetus and the fetal brain and skeleton (the only organs available) were represented as spherical or cylindrical shapes. The induced current densities and the electric fields were estimated in the fetus from applied electric and magnetic fields, separately, at 50 Hz. The magnetic field was oriented to obtain a front-to-back, lateral and top-to-bottom exposure (B_front_, B_lat_ and B_top_ in the following indicated as “classical” orientations) on the pregnant body. In [21], a 30-week pregnant woman model was developed, in which only the fetal skeleton and soft tissues could be distinguished. Induced electric current densities in the fetal soft tissues and in the fetal CNS tissues have been calculated for an exposure to 100-μT homogeneous magnetic fields at 50 Hz in the three classical exposure scenarios and for a vertically-oriented 5-kV/m electric field. In [22], a model of a pregnant woman at 30 weeks of pregnancy was developed in which no organs could be distinguished in the fetus. The exposure to a homogeneous magnetic field in the front and lateral exposure (B_front_ and B_lat_) and to a vertical electric field at 50 Hz has been analyzed, separately. In [23], seven pregnant female models have been analyzed, calculating the induced current densities in the fetal brain and body from applied magnetic fields at 50 Hz. Finally, in [24], the fetal exposure at 3, 7 and 9 months of gestational age (GA) to uniform magnetic fields (MF) at 50 Hz has been studied by means of advanced numerical models of pregnant women. In this work, the induced electric fields and electric current densities have been assessed in each fetal tissue at each GA (15, 17 and 26 tissues at 3, 7 and 9 months GA, respectively), when the **B**-field is oriented to obtain the previously-mentioned classical exposure conditions on the pregnant body. Different from the studies previously described, in which there was a lack of accuracy at the level of tissues and organs in the fetal models, in [24], a complete description of the fetal exposure is given through a tissue-specific analysis; however, also in this work, only three exposure conditions have been taken into account. Overall, this previous literature gave an almost exhaustive picture of the fetal exposure only for uniform magnetic fields oriented in three classical orthogonal directions. However, the direction of the field could be different from these ones in a realistic exposure scenario, and the analysis of only these classical orientations could be insufficient to find the worst-case exposure condition for each specific fetal tissue. To tackle this issue, in this study, the variability of fetal exposure due to **B** orientation has been investigated in terms of the assessment of the induced electric field (**E**) in each fetal tissue at 3, 7 and 9 months GA. In order to overcome the computational effort of the deterministic dosimetry, an analytical approximation of **E** in each fetal tissue has been developed by making use of the PC theory. Then, the PC expansions of **E** built for each tissue have been used to estimate the variability of **E** under different exposure conditions. Furthermore, the maximum induced electric field, found in each fetal tissue among all **B** orientations analyzed, has been identified and compared to the basic restrictions of the International Commission of Non-Ionising Radiation Protection (ICNIRP) Guidelines 2010 [3] for the general public at 50 Hz. Finally, an analysis of the distribution of **B** field orientations, which induce high electric fields (≥70% of the maximum **E**) in the fetus whole-body at each GA, has been also carried out.

## 2. Material and Methods 

### 2.1. Polynomial Chaos Expansion of the System Output Y 

Polynomial chaos (PC) is a spectral method used to assess how the variability of the input random parameters **X** influences the system output Y. In detail, the system output is approximated by a suitable finite-dimensional polynomial basis **Ψ(X)** of size *P* [26] as follows in (1):

(1) $$\text{Y}=M\left(X\right)=\overset{P-1}{\underset{j=0}{\sum }}a_{j\;}\psi_{j}\left(X\right)+e$$

where *M* is the model function, *a_j_* are the coefficients of the PC expansion, $\psi_{j}(X)$ are the polynomials of the basis **Ψ(X)** and *e* is the truncation error. Following the approach described in [13] and applied in [26], Y can be modelled as in (1), under the hypothesis that Y has a finite variance (E [Y^2^] < ∞). Furthermore, the input parameters **X** are supposed to be independent variables characterized by the joint probability density function (pdf) *f_X_*, expressed as in (2):

(2) $$f_{X}=\overset{K}{\underset{i=1}{\prod }}f_{x_{i}}$$

where *K* is the size of the input random vector **X** and $f_{x_{i}}$ is the probability density function associated with each input random parameter *x_i_*. This pdf *f_X_* is necessary to define the polynomial basis **Ψ(X)** used to build the PC expansion (1). Indeed, considering the independence of the input parameters *x_i_* stated above, each polynomial ψ(X) belonging to **Ψ(X)** can be represented as in (3):

(3) $$\psi \left(X\right)=\overset{K}{\underset{j=1}{\prod }}\pi_{\alpha_{j}}\left(x_{j}\right)=\pi_{\alpha_{1}}\left(x_{1}\right)\;\times \ldots ..\times \pi_{\alpha_{K}}(x_{K})$$

where $\pi_{\alpha_{j}}$ is a suitable family of polynomials orthogonal with respect to the pdf $f_{x_{i}}$ of each input parameter *x_i_* and $\alpha_{j}$ represents the maximum degree of the polynomials in $\pi_{\alpha_{j}}$. $\alpha =\left\{\alpha_{1},\ldots .,\alpha_{K}\right\}$ is the vector of the degrees $\alpha_{j}$. Only the combinations of the $\alpha_{j}$, such that $\left|\alpha \right|=|\alpha_{1}+\ldots +\alpha_{K}|\leq p$, where *p* is the maximum accepted degree of the polynomial ψ(X), are suitable to be used to build the polynomials ψ(X). The choice of the maximum degree *p* is arbitrary [11,27] and depends on the desired accuracy of the PC expansion. Table 1 shows the correspondence between some classic pdfs and their corresponding orthogonal polynomials [15].

**Table 1:** Correspondence between classic probability density functions (pdfs) and families of orthogonal polynomials.

Probability Density Function	Support *	Polynomial π
**Gaussian**	ℝ	Hermite
**Uniform**	(−1,1)	Legendre
**Gamma**	(0,+∞)	Laguerre
**Chebyshev**	(−1,1)	Chebyshev
**Beta**	(−1,1)	Jacobi

* The support is the set of points where the pdf is not zero-valued.

The size of the polynomial basis **Ψ(X)** is indicated as *P* in (1) and is a function of *p* and *K*:

(4) $$P=\left(\begin{array}{c} p+K\\ p \end{array}\right)$$

**Figure 1:**
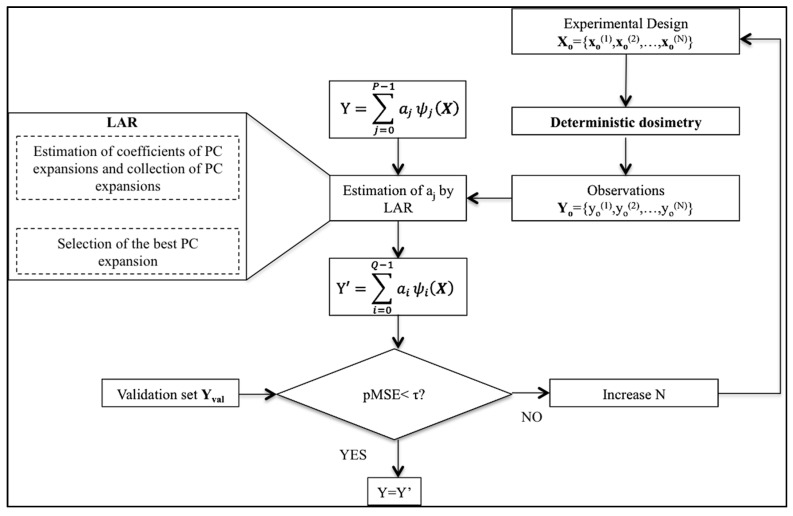
Schematic of the complete polynomial chaos (PC) procedure with a zoom of the “estimation of a_j_ by LAR” block on the left. LAR, least angle regression.

Figure 1 is a summary of the procedure adopted to perform the next steps to build and validate the PC expansion once the basis **Ψ(X)** has been built. 

The first step consists of optimizing Equation (1) by choosing the best combination of coefficients *a_j_* and the corresponding polynomials ψ(X) in **Ψ(X)**. In the literature, several methods have been proposed to perform this optimization [26,28,29,30,31]. Among them, here, the least angle regression algorithm (LAR), introduced by Efron and colleagues [32] and adapted to PC theory in [26], has been applied. LAR relates to the classic model-selection method known as forward selection [33]. The algorithm consists of selecting the most suitable polynomials from the chosen basis **Ψ(X)** and then of calculating the coefficients *a_j_* by least-square regression with the aim to optimize the PC approximation of Y with respect to a series of N observations Y_o_ = {*y_o_^(1)^, y_o_^(2)^,…,y_o_^(N)^*} of Y. In this study, the N observations have been obtained by deterministic dosimetry applied to a random subset of N input vectors X_o_ = {*x_o_^(1)^, x_o_^(2)^,…,x_o_^(N)^*}, which is the “experimental design” block in the schematic of Figure 1. Once the observations Y_o_ have been calculated, the LAR algorithm generates a collection of PC expansions, in which the first PC expansion includes a single polynomial of the basis **Ψ(X)**, the second one includes two polynomials, and so on, until *m* polynomials have been included, with *m* = min(*P*, N-1). Among these *m* PC expansions, the optimal one Y’ (see Figure 1) is then chosen as the minimum of the *m* leave-one-out cross-validation (LOOCV) errors. The LOOCV approach was proposed by [34,35] and applied to assess the accuracy of PC expansions obtained by LAR in several studies, such as [17,26]. One should note that in the optimization phase, the LAR procedure could select as the best solution Y’ a PC expansion with a number of coefficients *a_j_* less than *P*. Hence, in the following, the number of coefficients of Y’ will be indicated as Q. In this study, a home-made Python script based on the open TURNS package [36] has been used for the implementation of the above-explained procedure.

### 2.2. Validation of the PC Expansion

Once the PC expansion has been obtained (Y’ in Figure 1), it has to be validated. In this study, the validation consists of the estimation of the residual error with respect to a validation set **Y**_val_, different from the set **Y**_o_ previously used to build Y’. **Y**_val_ has been always estimated by deterministic dosimetry on an experimental design **X**_val_, randomly selected and different from **X**_o_, and has size S = N/2. The residual error is the percentage mean square error (pMSE) defined as in (5):

(5) $$\text{pMSE}=\frac{\sum_{i=1}^{S}{\left(\frac{Y_{val\left(i\right)-}Y_{i}^{\prime }}{Y_{val(i)}}\right)}^{2}}{S}*100$$

The entire PC procedure should be repeated increasing the size N of the experimental design and changing the maximum degree *p* of the polynomials ψ(X) until the achievement of a pMSE below a given threshold τ. This means that the LAR procedure is restarted with a higher number N of observations and, for each set of observations, changing the degree *p* of the polynomials (not shown in Figure 1) to obtain a new PC expansion Y’ (Figure 1). In this study, the threshold τ has been fixed to 0.5%.

### 2.3. Application of the PC Theory to the Analysis of the Fetal Exposure Varying B-Field Orientation

#### 2.3.1. Definition of the Input Random Vector **X** and the System Output Y

Stochastic dosimetry has been implemented in this study to quantify the influence of the **B**-field orientation at 50 Hz on the fetal exposure at different stages of pregnancy by means of the polynomial chaos theory. In this study, the input random vector **X** is made by *K* = 2 parameters: the spherical angles theta (θ) and phi (φ), which characterize the **B**-field orientation. These two variables are independent and supposed to be uniformly distributed over (0,180°) and (−180°, 180°), respectively. Hence, according to Table 1, each polynomial ψ(X) belonging to the basis **Ψ(X)** is made of Legendre polynomials, orthogonal with respect to the uniform distribution.

The system output Y consists of the 99th percentile value of the root mean square of **E** averaged over a 2 × 2 × 2 mm^3^ cube in each fetal tissue (E_99th_). Indeed, as the ICNIRP Guidelines 2010 [3] suggest, the induced **E** has to be calculated as a vector average in a small contiguous tissue volume of 2 × 2 × 2 mm^3^ in each specific fetal tissue, and the relevant value to be compared with the basic restriction is the 99th percentile value of the root mean square of **E** in each tissue. Therefore, this is the metric modelled by polynomial chaos. 

#### 2.3.2 The Observation Set **Y**_o_

In order to apply the LAR algorithm described in Subsection 2.1, we need N observations of E_99th_ for each fetal tissue. In this study, these observations have been obtained by deterministic dosimetry applied to an input vector X_o_, made of N pairs of angles [θ, φ], generated through a quasi-Monte Carlo method [37], based on Sobol’s function [38] and representing N different orientations of the **B**-field. 

**Figure 2:**
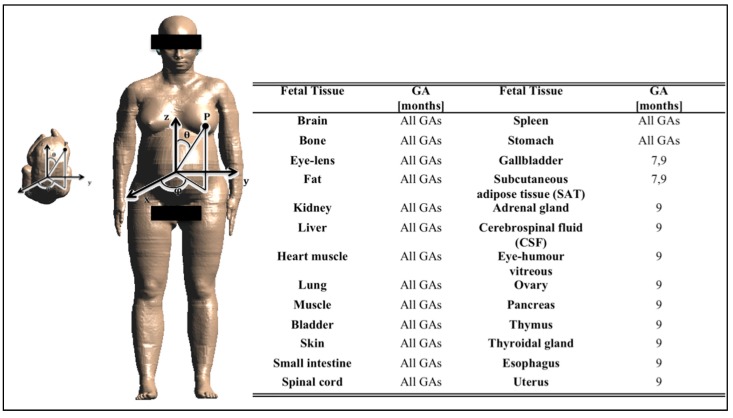
On the left: pregnant woman model at 9 months gestational age (GA) with the Cartesian reference system x,y,z and the spherical coordinates θ, φ. The fetus image is also reported to present its position in the pregnant woman’s womb. On the right: table with the fetal tissues at each GA.

The procedure of electromagnetic simulation used in this study has been already proposed by the authors in [24]. Briefly, three high-resolution pregnant woman models at 3, 7 and 9 months of gestational age (GA), based on the model “Ella” of the Virtual Family [39] and made available for research purposes through the Foundation for Research on Information Technologies in Society (IT'IS), were used. An example of a pregnant woman model at 9 months GA is provided in Figure 2, where also the Cartesian reference system x, y, z and the spherical coordinates θ, φ are represented. The mass of the fetus models at the three gestational ages is 15 g, 1.7 kg and 2.7 kg, respectively. Due to the formation of the organs at different stages of pregnancy, the three fetus models distinguish different tissues (as reported in the table in Figure 2). More details about the construction of the models are provided in [40].

Simulations were conducted using the Magneto Quasi-Static low frequency solver implemented in the simulation platform SEMCAD X v. 14.8 (Schmid & Partner Engineering AG, Zürich) [41]. The pregnant woman tissues (including fetus) at 7 and 9 months GA were discretized with a grid resolution of 1 mm, whereas at 3 months GA, a grid resolution of 0.3 mm was chosen in order to discretize the fetal skin. The conductivities of most of the woman tissues were assigned according to the data available in literature [42,43]. The fetal conductivity values were assigned as the adult ones apart from brain, bone and fat tissues, in which there is evidence of higher water content in the fetus than in the adult [7]. Details of the chosen conductivity values are described in [24]. The pregnant models were exposed to a perfectly homogeneous **B**-field at 50 Hz. The root mean square amplitude of the uniform **B**-field was set to the reference levels of the ICNIRP 2010 [3] for the general public at 50 Hz, which is 200 μT.

### 2.4. Analysis of the Fetal Exposure

Once the PC expansion Y of E_99th_ for each fetal tissue has been achieved, the statistical moments of the first and second order, *i.e*., the mean μ and the variance $\sigma^{2}$, of E_99th_ in each fetal tissue have been analytically derived from the PC coefficients following a method proposed and validated by Blatman and colleagues in [26]. In detail, the mean and the variance are expressed as in (6) and (7):

(6) $$\mu =a_{0}$$

(7) $$\sigma^{2}=\overset{Q-1}{\underset{j=1}{\sum }}{a_{j}}^{2}$$

As previously said, Q is the number of coefficients in the PC expansion of E_99th_ for each fetal tissue.

The coefficient of variation (CV), expressed as the ratio of the standard deviation to the mean, has been then calculated to discuss the influence of the **B**-field orientation on the fetal exposure of each tissue and at each GA. 

Moreover, through the PC expansions, E_99th_ in each tissue was computed for 10,000 **B** orientations, defined as pairs of angles [θ, φ] randomly selected, and the maximum among, 10,000 E_99th_ (mE_99th_), has been compared to the limits (E_lim_) indicated by the basic restrictions of the ICNIRP Guidelines 2010 [3] for the general public at 50 Hz. These limits are 0.02 V/m for the central nervous system (CNS) tissues of the head and 0.4 V/m for all of the other tissues of the head and body. For convenience, the percentage ratio between mE_99th_ and E_lim_ in each fetal tissue (in the following identified as worst-case scenario WS%) is used to provide quantitative information about the worst exposure scenario with respect to the permitted values and is defined as:

(8) $$\text{WS\%}=\frac{\text{m}E_{99th}}{E_{lim}}*100\;$$

Finally, an analysis of the **B** orientations, which induce E_99th_ ≥ 70% mE_99th_ of the fetus whole-body at each GA, has been performed. The threshold of 70% was chosen, because it corresponds to an amplitude reduction of 3 dB with respect to the maximum value [44].

## 3. Results and Discussion

### 3.1. Validation of the PC Expansion in Each Fetal Tissue 

In order to choose the best PC expansion of E_99th_ in each fetal tissue, the validation procedure described in the Material and Methods Section has been performed, estimating the residual error, expressed as pMSE (see Equation 5), between a validation set **Y**_val_ and several PC expansions of E_99th_ built from sample sets **Y**_o_ of increasing size N and changing the maximum degree *p* of the polynomials ψ(X). The error pMSE was found to be lower than the error threshold τ, fixed at 0.5%, when the PC expansions were built using N = 300 observations and a degree *p* = 15. Therefore, this number of observations and the same degree *p* were used in this study to build all of the PC expansions.

### 3.2. Estimation of the Statistical Moments

**Figure 3:**
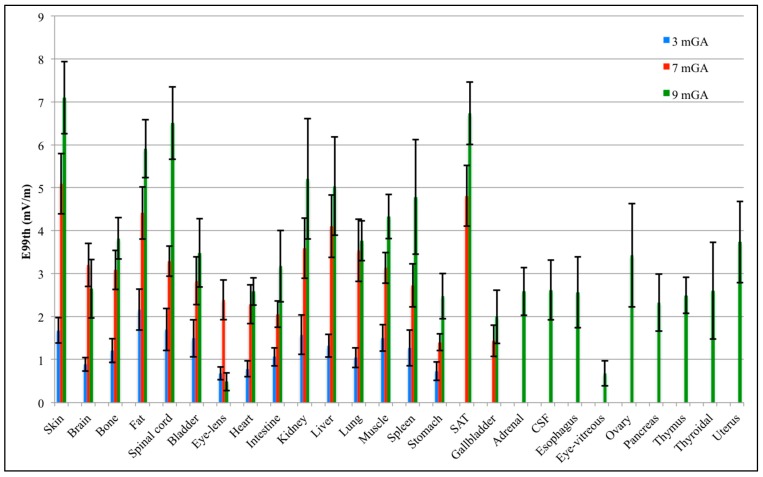
Mean value (bar) and standard deviation (whiskers) of E_99th_ in each fetal tissue at 3, 7 and 9 months GA.

Figure 3 represents the mean μ and the standard deviation σ of E_99th_ for each fetal tissue at 3, 7 and 9 months GA estimated by PC coefficients. This graph represents the effect of the variation of **B** orientation on E_99th_ for each fetal tissue.

As already observed in [24], the mean of E_99th_ across the **B** orientations is greater with GA due to the increase in size of fetal organs and tissues. Moreover, considering only the common tissues at all GAs, the tissues with the highest mean E_99th_ are the fetal skin, fat, liver, spinal cord and kidney, with a mean value up to 7.10 mV/m at nine months GA in the skin tissue. At three months GA, the highest mean E_99th_ has been found in the fetal fat (around 2.16 mV/m), while the lowest exposed tissue is the eye-lens, with a mean E_99th_ of about 0.68 mV/m. The standard deviation has a maximum of 0.49 mV/m in the spinal cord and a minimum of 0.15 mV/m in the eye-lens and brain. At seven months GA, the highest exposed tissue, in terms of mean E_99th_, is the fetal skin (5.09 mV/m). Furthermore, fetal brain, bone, spinal cord and muscle present similar mean E_99th_ values of about 3 mV/m, whereas the fetal stomach and gallbladder, which are the lowest exposed tissues, show mean E_99th_ values around 1.40 mV/m. The highest standard deviation is 0.73 mV/m in liver, and it is around 0.70 mV/m in skin, kidney, lung and subcutaneous adipose tissue (SAT), whereas the lowest variation of 0.19 mV/m is in the stomach tissue. At nine months GA, some tissues present similar mean E_99th_ (e.g., brain, heart, adrenal, CSF, esophagus and thyroidal) of about 2.60 mV/m, and the highest standard deviation is up to 1.40 mV/m in kidney. 

**Figure 4:**
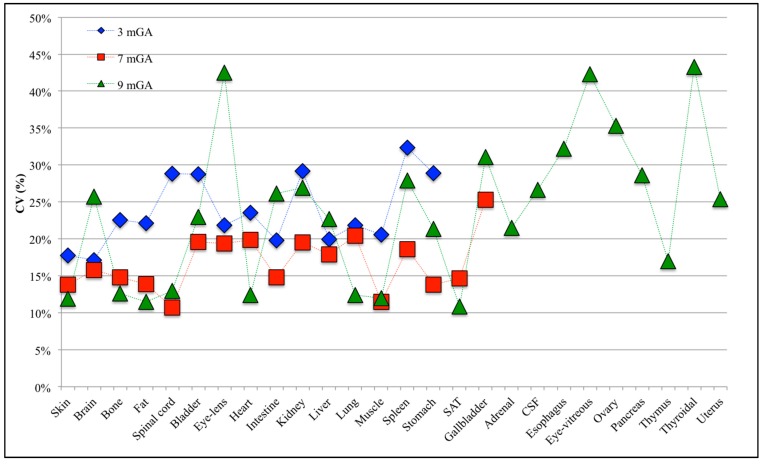
Coefficient of variation (CV) for each fetal tissue at 3, 7 and 9 months GA.

The variability of E_99th_ values, due to the different **B**-field orientations, is more clearly highlighted in Figure 4 in terms of CV coefficients for the various fetal tissues at all GAs. CV has been found always higher than 10% at all GAs. Fetal spleen, gallbladder and thyroidal gland present the highest CV at 3, 7 and 9 months GA with values up to 32.37%, 25.30% and 43.25%, respectively. CVs higher than 30% and up to 35% have been also observed in the fetal gallbladder, esophagus and ovary at nine months GA. 

Analyzing the CV in the fetal tissues that are common to all GAs, all of them, apart from the fetal brain, eye-lens, intestine and liver, are more influenced by the orientation of the incident **B**-field at three months GA than at the other stages of pregnancy, with CV up to three-times higher in the spinal cord at three months of GA than for the other two GAs. This difference among GAs could be related to computational uncertainty due to the different grid resolution at three months GA (*i.e.*, of 0.3 mm) with respect to seven and nine months GA (*i.e.*, 1 mm), adopted for the application of the deterministic dosimetry to estimate the observations **Y**_o_ necessary to build the PC expansion (see Figure 1 above). However, Liorni and colleagues [24] demonstrated that the uncertainty of the grid resolution affects the estimated E_99th_ by no more than 5%; thus, the high CV value at three months GA is probably linked to the specific shape and small size of the involved tissue. Among the tissues that are common to all GAs, fetal bladder, eye-lens, kidney and spleen always present CV almost equal or higher than 20%, whereas for the fetal bone, the maximum CV is 22.5% at three months GA, with values of 14.8% and 12.59% at seven and nine months, respectively. 

### 3.3. Analysis of the Fetal Exposure Respect to the Limits

The maximum E_99th_ (mE_99th_) in each fetal tissue has been found over 10,000 different **B** orientations and compared with the exposure limits (E_lim_) proposed in the ICNIRP Guidelines 2010 [3]. The analysis has been carried out considering (i) the central nervous system (CNS) tissues of the head and (ii) all of the other tissues of the head and body, separately. 

The WS% ratio, defined in (8), in the CNS tissues of the head has been found equal to 23.50% at nine months GA, 23% at seven months GA and 7.75% at three months GA, meaning that mE_99th_ is notably lower than the ICNIRP limits at all GAs. 

**Figure 5:**
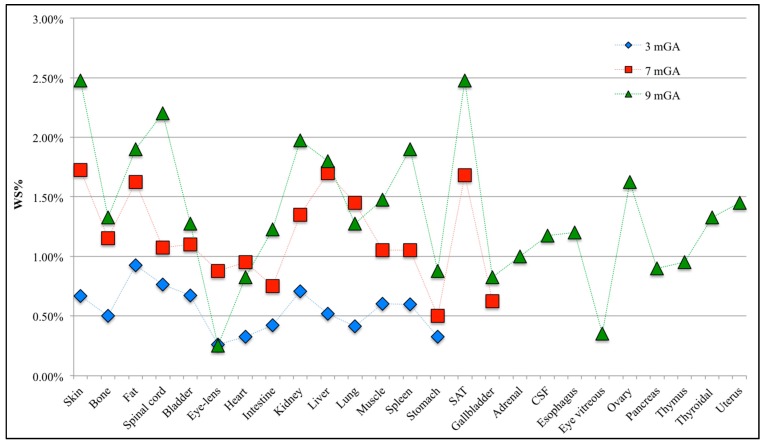
Worst-case scenario (WS%) in all the tissues of the head (excluding the CNS) and body for the mE_99th_ found among the 10,000 values of E_99th_ calculated from the PC expansion, changing randomly the **B** orientations.

Figure 5 represents the WS% for all of the other tissues of the head and body at each GA. Furthermore, in this case, the exposure resulted in being well below the limit of 0.4 V/m, with a WS% ratio always lower than 3%. 

**Figure 6:**
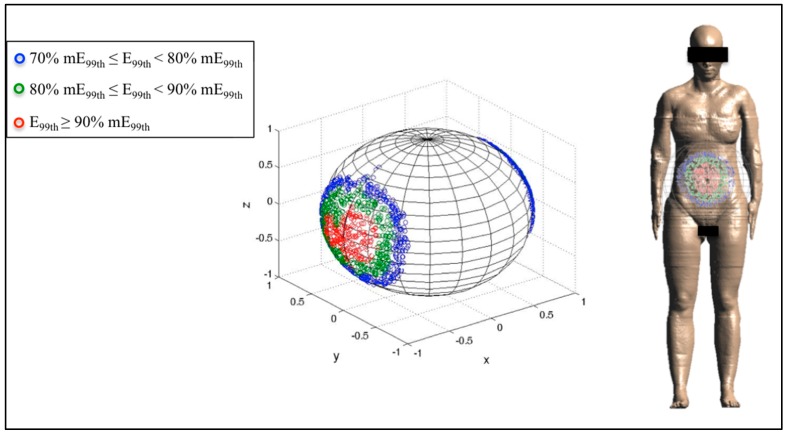
Distribution of **B**-field orientations on a unitary sphere represented in the pregnant woman’s reference system x,y,z (Figure 2), which determines, on the fetus whole-body at three months GA, induced electric field E_99th_ in the ranges 70% mE_99th_ to 80% mE_99th_ (blue circles), 80% mE_99th_ to 90% mE_99th_ (green circles) and higher than 90% mE_99th_ (red circles), respectively. These **B** orientations have been also indicated with respect to the reference system centered on the pregnant woman adopted in this study.

**Figure 7:**
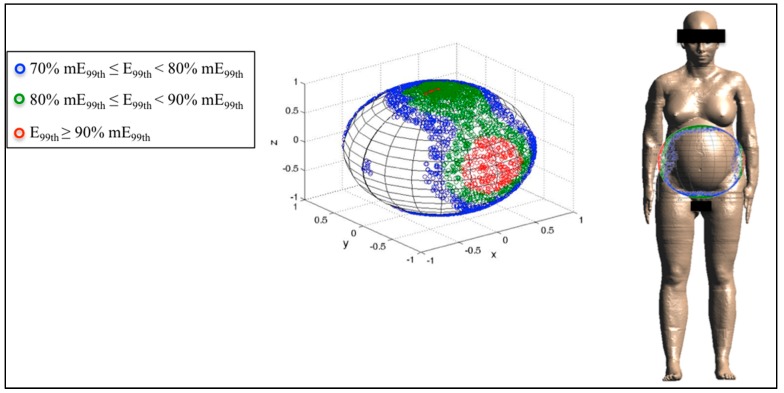
Distribution of **B**-field orientations on a unitary sphere represented in the pregnant woman’s reference system x,y,z (Figure 2), which determines, on the fetuswhole-body at seven months GA, induced electric field E_99th_ in the ranges explained in Figure 6. The **B** orientations have been also indicated with respect to the reference system centered on the pregnant woman adopted in this study.

**Figure 8:**
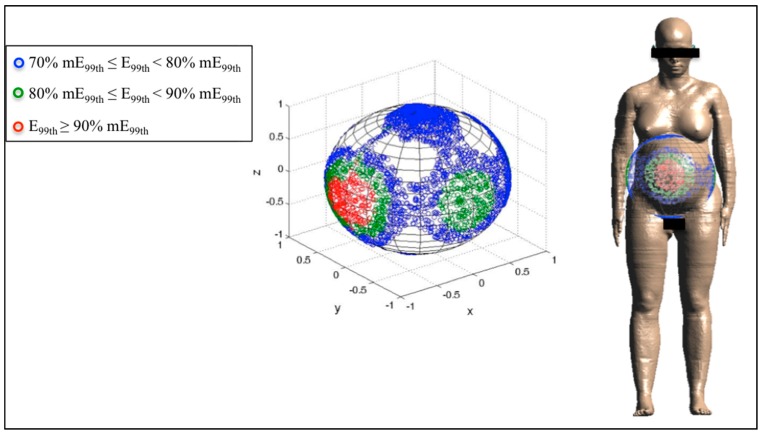
Distribution of **B**-field orientations on a unitary sphere represented in the pregnant woman’s reference system x,y,z (Figure 2), which determine, on the fetus whole-body at nine months GA, induced electric field E_99th_ in the ranges explained in Figure 6. The **B** orientations have been also indicated with respect to the reference system centered on the pregnant woman adopted in this study.

Finally, Figure 6, Figure 7 and Figure 8 represent the distribution on a unitary sphere of those **B** orientations, among the 10,000 analyzed, which induce E_99th_ higher than 70% of mE_99th_ in the fetus whole-body at 3, 7 and 9 months GA. In detail, in the figures, the blue circles represent the **B** orientations for which E_99th_ in the fetus whole-body is in the range from 70% to 79% of mE_99th_, the green circles in the range from 80% to 89% of mE_99th_ and the red circles all of the orientations of the **B**-field for which E_99th_ is higher than 90% of mE_99th_. 

At three months GA, mE_99th_ has been found for both the front-to-back and back-to-front exposure with respect to the pregnant woman body (Figure 6). The solid angle of the sphere under the region E_99th_ ≥ 90% mE_99th_ is equal to 0.27 steradians (sr) with respect to the front side of the pregnant woman and symmetrically also to the back side. Furthermore, the other **B** orientations, which determine the three ranges of values of E_99th_ indicated above, are always located around the **B** orientation for which E_99th_ is maximum, and the total solid angle under these three areas analyzed is equal to 1.43 sr.

At seven months GA, mE_99th_ is induced by the lateral exposure (both right-to-left and left-to-right) with respect to the pregnant woman body (Figure 7). Furthermore, in this case, the region of the sphere with E_99th_ ≥ 90% mE_99th_ is determined by a solid angle of 0.26 sr with respect to each lateral side of the pregnant woman.

However, different from the previous stage of pregnancy, values of E_99th_ ≥ 80% mE_99th_ have been found for **B** orientations significantly far from the lateral exposure and closer to the top and bottom exposure (as can be observed in Figure 7). The solid angle under the sphere, which represents this last region, is 0.48 sr for the top side and symmetrical for the bottom one.

Finally, at nine months GA, mE_99th_ has been found for the front-to-back and back-to-front exposure as at three months GA (Figure 8). The region of the sphere in which **B** orientations induce E_99th_ ≥ 90% has a solid angle of 0.34 sr with respect to the front side and symmetrically for the back side of the pregnant woman. However, as previously said for the seven-month fetus, there are several orientations of the **B**-field around the lateral exposure, which induce electric fields higher than 80% and up 89% of the maximum. The solid angle relative to this region is 0.24 sr for each lateral side of the pregnant woman.

In general terms, the location of the three regions introduced above depends also on the position of the fetus in the pregnant woman’s womb. In this study, the variability of fetal exposure has been assessed considering the variation of the exposure configurations, *i.e*., the orientation of the incident **B**-field with respect to the pregnant woman’s body, whose morphology was unchanged. However, one should take into account that also the movements of the fetus in its mother’s womb would additionally contribute to varying the induced fields in the fetal tissues. In [24], the variability of the exposure due to the fetal posture was quantified on three pregnant woman models at three months GA, in which the fetal posture was changed among the most statistically significant at that stage of pregnancy. A detailed description of the various fetal positions during pregnancy is provided in [45]. The three months GA pregnant models were exposed to uniform **B**-field oriented with respect to the classical directions B_front_, B_lat_ and B_top_. In this condition, the maximum variation of E_99th_ induced in the whole-body, due to the fetal posture, was found to be up to 18%.

## 4. Conclusions 

In this study, stochastic dosimetry in the form of polynomial chaos theory has been used to assess the variability of fetal exposure to low-frequency EMF in a realistic exposure scenario. Up till now, very few studies applied PC theory to assess the LF-EMF exposure, always disregarding the analysis of the fetuses. On the contrary, in this work, the fetal exposure at different stages of pregnancy to a uniform magnetic field at 50 Hz as a function of the **B** orientation was assessed, by developing an approximation of the induced electric field E_99th_ in each fetal tissue by polynomial chaos and studying how the variability of **B** orientation influences E_99th_. Indeed, there is the necessity to close the gap of knowledge about possible worst-case exposure scenarios in fetal tissues different from the ones obtained for the classical orientations of the magnetic field (*i.e.*, B_front_, B_lat_ and B_top_) that have so far been analyzed in the previous literature by deterministic dosimetry. 

The PC approach resulted in an efficient method to build accurate approximation of E_99th_ in each fetal tissue (from the validation procedure, a maximum pMSE of 0.48% was obtained across all fetal tissues) and to perform a complete analysis of the fetal exposure at a lower computational cost than the one required by the deterministic dosimetry. Indeed, the number of observations **Y**_o_ (in this case N = 300), calculated by deterministic dosimetry and necessary for building the PC expansion of E_99th_, was much lower than the number of possible variations of the input parameters under study, lowering therefore the computational cost. Once the observations **Y**_o_ have been collected, the construction of the PC expansion by means of the home-made Python script and the calculation of the E_99th_, changing **B** orientations, require only a few minutes. 

However, in general terms, one should take into account that the choice of the number of observations **Y**_o_ is a critical issue and depends on the compromise between the computational cost necessary for their estimation and the precision that can be considered acceptable for the specific situation under investigation. Indeed, there could be cases where the estimation of the observations is complex and time consuming and the vector **Y**_o_ has to be reduced at the expense of the accuracy of the stochastic modelling.

In this study, the analysis of the fetal exposure showed that, considering the fetal tissues common for all GAs, the fetal skin, fat, spinal cord, liver and kidney were on average the most exposed tissues changing the orientation of **B**. Moreover, in these same tissues, in almost all cases, E_99th_ was found more influenced by the **B**-field orientation at three months GA than at the other stages of pregnancy. This could probably be due to the shape and size of the tissues and the organs at that GA. 

The effect of the **B**-field orientation cannot be considered negligible, considering that a variability of E_99th_ has been estimated ranging from 10% to 43% in terms of CV across all fetal tissues and GAs. On the other side, even analyzing the maximum E_99th_ found across 10,000 **B** orientations, the fetal exposure resulted in being largely lower than the basic restriction of the ICNIRP 2010 [3] for the general public at 50 Hz for both the CNS tissues of the head and for all of the other tissues of the head and body. Indeed, a maximum WS% of 23.50% was found in the CNS tissues of the head at nine months GA, whereas it has been always found lower than 3% in the evaluation of all of the other tissues of the head and body. Therefore, the induced electric fields were well within the ICNIRP basic restrictions in all cases for exposures at the general public reference levels, disregarding the orientation of **B**. 

A further analysis on the 10,000 **B** orientations showed that the highest induced electric field in the fetus whole-body at all GAs was found for the classical orientations B_front_ at three and nine months GA and B_lat_ at seven months GA, in agreement with the results already found in [24]. However, the induced E_99th_ ≥ 90% mE_99th_ have been also found for other **B** orientations around the classical worst-case exposure condition, which describe a region that is quite similar in size at all GAs. 

Furthermore, at seven and nine months GA, there are some **B** orientations, different from the ones that induce the maximum electric field in the fetus whole-body, which determine E_99th_ in the range 80% to 89% of mE_99th_. This means that with increasing the gestational age, there is a major dependence of the fetal exposure to the orientation of the incident **B**-field, which is able to induce a high electric field in the fetus for several orientations.

In conclusion, stochastic dosimetry allowed assessing the influence of the **B**-field orientation at 50 Hz on the fetal exposure by developing a PC expansion of E_99th_ in each fetal tissue. This study has demonstrated that polynomial chaos is a powerful tool to evaluate the influence of the variation of the input parameters in a realistic exposure scenario, overcoming the problems of the high computational costs faced by deterministic dosimetry. Therefore, it could be used in the future for the analysis of the variations of other parameters that influence the human exposure to EMF. 

The variation of **B** orientation significantly influences the fetal exposure in some specific tissues at all GAs. However, though there is a significant variation of exposure, the highest levels of induced electric fields in all tissues are always found within the limits of the ICNIRP 2010 [3]. Finally, several directions of **B**, far from the exposure conditions that determine the maximum of E_99th_, have been found to induce a high electric field (from 80% to 89% of the maximum) especially at seven and nine months GA. Therefore, the polynomial chaos approach also permitted accurately studying the influence on the maximum exposure due to several **B** orientations. 
